# Treatment selection and functional outcomes in motor cortex metastases

**DOI:** 10.1007/s11060-026-05758-2

**Published:** 2026-08-17

**Authors:** Luisa Mona Kraus, Maria Goldberg, Paul Simon Backhaus, Carolin Albrecht, Denise Bernhardt, Stephanie E. Combs, Chiara Negwer, Bernhard Meyer, Arthur Wagner

**Affiliations:** 1 Department of Neurosurgery, University Hospital of Technical University Munich, School of Medicine and Health, Ismaninger Str. 22, 81675 Munich, Germany; 2 Department of Radiation Oncology, University Hospital of Technical University Munich, School of Medicine and Health, Munich, Germany; 3 Institute of Radiation Medicine (IRM), Helmholtz Zentrum München (HMGU), Neuherberg, Germany; 4 German Consortium for Translational Cancer Research (DKTK), Partner Site Munich, Munich, Germany

**Keywords:** Brain metastases, Motor cortex, Microsurgical resection, Stereotactic radiosurgery

## Abstract

**Background:**

Brain metastases are associated with a poor prognosis. While systemic therapy primarily determines overall survival, surgical cytoreduction may improve local disease control. Preserving neurological function and independence is particularly important in patients with limited life expectancy. However, surgery is often avoided for metastases located in eloquent brain regions. This study evaluates treatment selection and functional outcomes in patients with metastases involving the primary motor cortex.

**Methods:**

We conducted a retrospective single-center analysis of patients treated for central motor eloquent brain metastases. Primary endpoints were motor function and KPS at discharge and at 6-month FU. Neurological change was categorized as improvement, stability, or deterioration in Medical Research Council (MRC) grade. Secondary variables included intracranial tumor burden, extracranial disease, prior systemic therapy, disease duration, which were used for risk stratification. Comparative analyses were performed using Wilcoxon rank-sum test, Fisher’s exact test, multivariable logistic, and Firth regression.

**Results:**

A total of 152 patients (79 male, 73 female; mean age 65.5 years) were eligible. Treatment comprised primary surgery in 109 patients, definitive stereotactic radiotherapy in 24, salvage surgery after radiotherapy in 17, and best supportive care in two. In single metastases with higher volume surgery was favored, whereas extracranial disease, prior systemic therapy, and longer disease duration were associated with allocation to radiotherapy. At discharge improvement rate was 13% (MSR) versus 25% (SRT) (*p* = 0.400), at 6-month FU improvement was 45% in the MSR and 9% in the SRT cohort (*p* = 0.063), while longitudinal analysis showed significant improvement in median MRC grade only in the surgery cohort.

**Conclusion:**

Surgery was not associated with inferior functional outcomes and even resulted in greater motor improvement at FU. These findings support surgery as a feasible treatment option, including for selected high-risk patients, while stereotactic radiotherapy seems to remain an important alternative for frailer patients with longer disease histories.

**Supplementary Information:**

The online version contains supplementary material available at https://doi.org/10.1007/s11060-026-05758-2.

## Introduction

Brain metastases (BM) are the most common intracranial tumors and still associated with a generally poor prognosis [1]. Indications for surgical interventions in BM – except in emergency situations – are typically discussed in a multidisciplinary setting. Given that most patients with BM ultimately die of the systemic disease rather than the consequences of a single metastasis, this remains a key consideration in treatment planning [2]. In this context, the role of microsurgical resection (MSR) versus stereotactic radiotherapy (SRT) must be carefully weighed [3]. Current clinical practice favors MSR when histopathological confirmation is required or when an immediate therapeutic effect is desired. This includes large BM with mass effect or lesions located in eloquent areas causing neurological deficits [4, 5]. Even in patients with multiple metastases, resection of the symptomatic index lesion may reduce seizure burden and improve functional status although a survival benefit remains debatable [6–9]. Cytoreductive surgery has also been shown to be feasible in recurrent BM [10]. However, neurological sequelae remain the most feared complication of resection of eloquent BM. In addition, surgery may increase the risk of leptomeningeal dissemination, whereas adjuvant radiotherapy carries a risk of radiation necrosis, both of which can independently compromise neurological function [11, 12]. In particular, preoperative radiotherapy and systemic chemotherapy have been associated with an increased risk of postoperative motor deficits in centrally located BM [13]. While en-bloc resection is generally preferred due to its lower risk of leptomeningeal disease spread and local recurrence [11, 12], it may not always be achievable – especially in eloquent areas [14]. Advances in surgical techniques, including intraoperative neuromonitoring, have improved safety [15–17]. Hence, MSR can lead to significant functional and neurological recovery in symptomatic patients [18, 19]. Nevertheless, comparisons between MSR and SRT in centrally located BM suggest a higher risk of motor impairment when surgery is performed [13, 16], whereas SRT may offer tumor control and a limited risk of motor deficits [20]. To optimize the safety of both surgical and radiosurgical treatments, advanced planning strategies such as transcranial motor stimulation (TMS) mapping have been introduced [21]. Incorporating TMS data into planning of adjuvant radiotherapy may allow for dose reduction and minimize treatment-related risks [22–25]. Despite these advances, patient-individual treatment selection remains complex and should be guided by a multidisciplinary expert panel. Although the relative merits of primary surgical versus radiation-based approaches in motor cortex BM have been debated both separately and in comparison, there is limited data on the underlying decision-making process. To contribute to this ongoing debate, we present our experience as a neurooncological center.

## Methods

We performed a single-center, retrospective data bank analyses of all patients treated either with MSR or SRT of eloquent central brain metastases.

The local ethics committee granted permission for this study (No.5626:12). Since a retrospective chart review was performed no additional patient consent was required.

Our inclusion criteria were:

- Diagnosis of a malignant tumor disease
- Availability of pre-treatment MRI
- Location of index metastasis in a) the precentral gyrus or b) the central sulcus on pre-treatment MRI
- Age > 18 years
- Documentation of multidisciplinary tumor board consensus
- Intended maximum safe resection in surgery group

Our exclusion criteria were:

- Postcentral metastases
- Surgical treatment by biopsy only
- Lack of treatment data

**Fig. 1:**
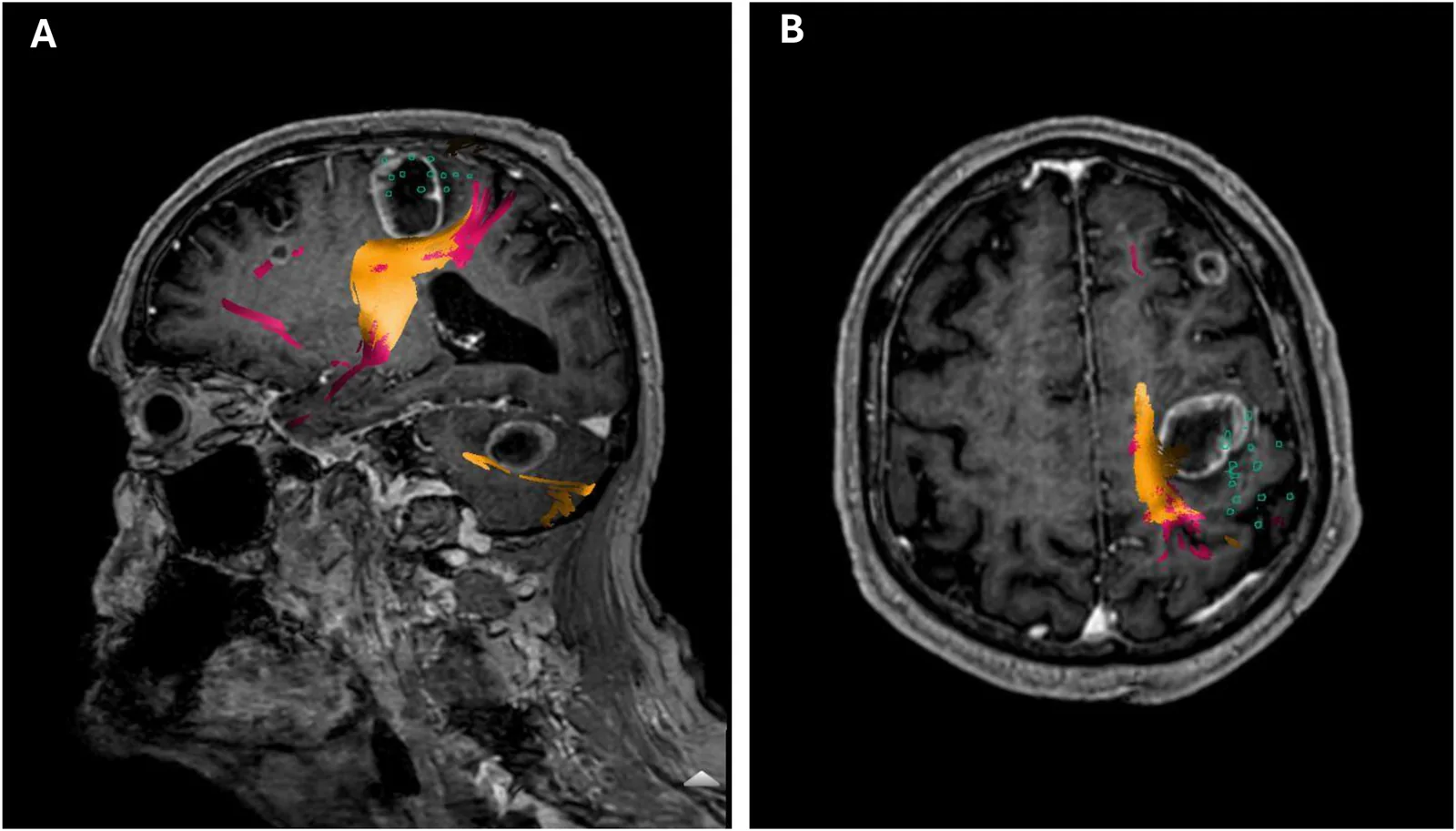
Visualization of the spatial relationship of a BM, the corticospinal tract (yellow), and the motor cortex area (green) using TMS mapping in sagittal (**A**) and axial (**B**) MR-imaging additional pink fibers depict sections of the arcuate fasciculus and inferior-frontal-occipital fasciculus used for speech mapping

All surgeries were performed with intraoperative electrophysiological monitoring of motor evoked potentials and intraoperative navigation. In patients being treated after 2021 and in selected cases before 2021 (*n* = 67 patients) a strict perioperative protocol that was based on preoperative TMS mapping and intraoperative TMS-based navigation was applied (Fig. 1). Starting 2023 intraoperative MRI was used for resection control in selected cases.

### Definition of independent baseline variables

Neurological performance was depicted by the worst single motor grade according to Medical Research Council (MRC) grading on the most severely affected limb on the contralateral side of the intracerebral lesion. Global functional status was quantified using the Karnofsky Performance Status [26]. Prior radiotherapy included SRT of the index metastasis as well as whole-brain radiotherapy (WBRT). Tumor volume was estimated using an ellipsoid approximation (π/6×A×B×C), based on the largest tumor diameters in three orthogonal dimensions on pre-treatment contrast-enhanced T1-weighted MRI and given in cm^3^ [27].

Median survival time was defined as survival from cancer diagnosis to last available FU or known date of death. In contrast, survival from treatment was defined as survival from either MSR or SRT to last available FU or known date of death. Charlson Comorbidity Index (CCI) was used to assess multimorbidity [28].

### Definition of high-risk patients in the MSR cohort

To identify patients with a potentially higher surgery risk profile, an exploratory post hoc score was developed. Score items were selected based on patient characteristics that were significantly more prevalent in patients allocated to SRT. All patients were scored dichotomically regarding intracranial tumor burden (solitary vs. multifocal), prior systemic treatment, prevalence of extracerebral metastases and time from cancer diagnosis to treatment. The median time from cancer diagnosis to treatment in this cohort was 98 days (IQR 7–947), thus one point was given if time was > 98 days. A cumulative score of 0–1 points was considered low risk, while a cumulative score of 2–4 was defined as high-risk (Supplementary Table 1).

Supplementary Table 1 Definition of risk score to identify high-risk patients A score of 0–1 was defined as low-risk, while a score of 2–4 was defined as high-risk. *Median time from cancer diagnosis to treatment in this cohort was 98 days

### Primary endpoints

Primary endpoints were postoperative neurological outcome at discharge or at first FU post radiotherapy respectively, ability to ambulate at discharge or at first FU post radiotherapy respectively and neurological outcome at six-month FU. A six-month FU was chosen to allow for sufficient data on long-term FU after completion of treatment in the context of a metastatic, life-limiting malignancy. Neurological improvement was defined as an increase in MRC of ≥1, deterioration was defined as a decrease in MRC of ≤1.

### Statistical analyses

GraphPad Prism (version 10.4.2) and R statistical software (version 4.6.0 (2026–04-24)) were used for statistical analyses and data interpretation. All data was assessed for Gaussian distribution. Medians or means were further used accordingly. Medians were compared using the Wilcoxon rank-sum test. Proportions were compared using the Fisher’s exact test. For contingency tables larger than 2 × 2 the Freeman-Halton extension of Fisher’s exact test was applied.

Multivariable logistic regression was used to identify factors associated with treatment allocation to MSR versus SRT. Candidate covariates included preoperative KPS, preoperative motor function, intracranial tumor burden, extracerebral disease, prior systemic therapy, volume and CCI. To assess predictors of neurological deterioration at 6-month FU, Firth logistic regression [29] was applied because of the limited number of deterioration events. Treatment modality was included as an adjustment variable, and pre-treatment motor function, preoperative KPS, and CCI were evaluated in separate parsimonious models to reduce overfitting.

Significance levels were * in *p* < 0.05, ** in *p* < 0.005 and *** in *p* < 0.001.

## Results

Between 2007 and February 2026 we treated 152 patients for eloquent central metastases at our neurooncological center. Median age was 65.5 (IQR 58–73) years; sex was nearly equally distributed (73 female (48%) and 79 male patients (52%)). The predominant cancer type was lung cancer (50%) followed by gastrointestinal tract cancer (GIT) (9.9%), breast cancer (9.3%), and malignant melanoma (7.9%) (Table 1).

**Fig. 2:**
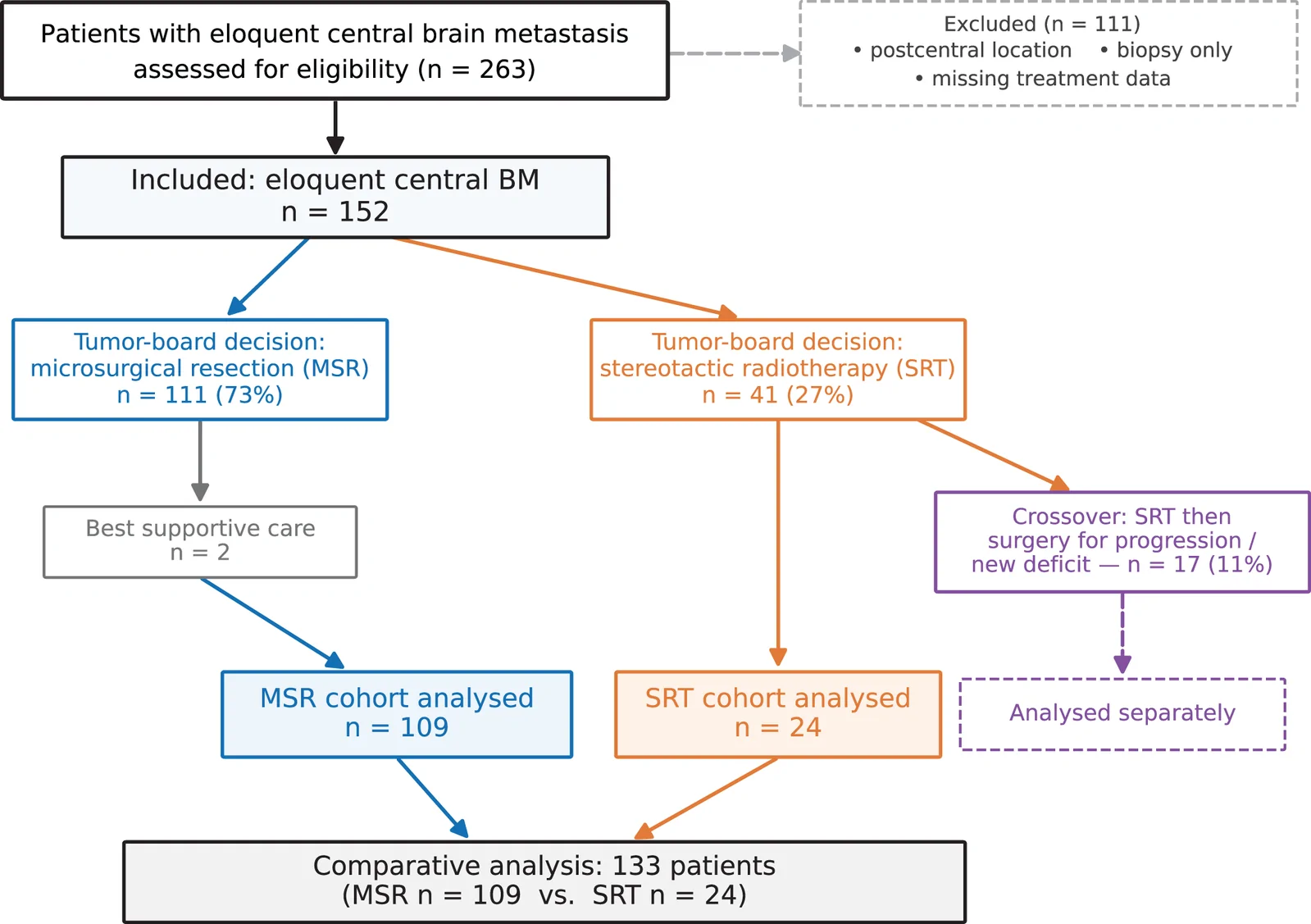
Cohort definition according to the interdisciplinary tumor board consensus when diagnosis of a central BM was made, 111 (73%) out of 152 eligible patients were considered suitable for MSR while 41 patients (27%) were scheduled for SRT. 17 patients (11%) received SRT but ended up undergoing additional surgery of the index metastasis

At the time of imaging diagnosis of eloquent central BM initial decision to perform MSR was made in 111 patients (73%) and to perform SRT in 41 patients (27%). Two patients (1.3%) deteriorated quickly and decision to proceed with best supportive care was made. Of the 41 patients who received radiation therapy of the index metastasis, 17 underwent subsequent surgery for tumor progression and/or neurological deterioration (Fig. 2). These 17 patients referred to as crossover cohort were thus excluded from sub-cohort comparison and described separately. Accordingly, the comparative analysis comprised 133 patients (109 MSR and 24 SRT); the remaining 19 patients consisted of 17 crossover patients, analysed separately, and two patients who received best supportive care.

**Table 1:** Clinical characteristics of patient cohort All continuous data are given as medians

Characteristic	N = 152^1^
Age at treatment	66 (56–75)
*Cancer type*	
Lung cancer	76 (50%)
Breast Cancer	14 (9.3%)
Malignant Melanoma	12 (7.9%)
GIT	15 (9.9%)
Urothelial	4 (2.6%)
GYN	2 (1.3%)
RCC	12 (7.9%)
NET	1 (0.7%)
Prostate	3 (2.0%)
Other	11 (7.3%)
CUP	2 (1.3%)
Number intracranial metastases	2 (1–3)
Hemorrhagic metastases	18 (12%)
Volume [cm^3^]	3 (1–6)
Extracerebral metastases	62 (41%)
BM first diagnosis of cancer	53 (35%)
Prior systemic treatment	66 (44%)
Time diagnosis to treatment[days]	400 (9–1,093)
Clinical performance pre	
KPS pre-treatment	80 (70–80)
Worst MRC pre-treatment	4 (3–4)
Seizure pre-treatment	47 (31%)
Ambulation pre-treatment	114 (76%)
CCI	0 (0–1)
Clinical performance post	
KPS post-treatment	80 (60–80)
Worst MRC post-treatment	4 (3–5)
Ambulation post-treatment	93 (66%)
Motor function post-treatment^ɑ^	
Stable	86 (58%)
Improved	32 (21%)
Worsened	31 (21%)
Length of hospital stay [days]	10 (7–16)
Survival from treatment [months]	3 (0–14)
Clinical performance 6-month FU^β^	
Worst MRC at 6 months	5 (4–5)
Motor function at 6 months	
Stable	34 (49%)
Improved	29 (41%)
Worsened	7 (10%)
Seizure at 6 months	8 (12%)
KPS at last FU	70 (40–80)

^1^Median (Q1–Q3); n (%)

^ɑ^n=149

^β^Of all data available at FU, n=70

GIT=gastrointestinal cancer, GYN=gynecological cancer, RCC=renal cell carcinoma, NET=neuroendocrine tumor, CUP=cancer of unknownprimary

### Baseline comparison of treatment groups

Median age was comparable in both treatment cohorts (66 years (IQR 57–75) in MSR vs. 63 years (IQR 55–75) in SRT; *p* = 0.200). While MSR favored lower intracranial tumor burden (*p* = 0.008); longer time from cancer diagnosis to treatment (*p* = 0.023), prior systemic treatment (OR 0.36, 95% CI 0.14–0.89; *p* = 0.038) and prevalence of extracerebral metastases (OR 0.24, 95% CI 0.09–0.60; *p* = 0.002) were associated with SRT (Table 2). Tumor volume was significantly larger in the MSR cohort (*p* = 0.002). The proportion of patients presenting with a first diagnosis of metastatic tumor disease did not differ significantly between treatment groups (MSR 41.3% vs. SRT 25.0%; OR 2.11, 95% CI 0.75–5.90, *p* = 0.220). There were no hemorrhagic metastases in the SRT cohort, yet this difference did not reach statistical significance (OR ∞, 95% CI 0.77-∞, *p* = 0.073). Median pre-treatment motor function differed between groups with MRC 4 (IQR 3–4) in the MSR cohort and MRC 4 (IQR 4–5) in the SRT cohort (*p* = 0.015). Preoperative Karnofsky-Index was 70 (IQR 60–80) in both cohorts (*p* = 0.940). Preoperative ambulation was preserved in 83.5% in the MSR cohort and 83.3% in the SRT cohort (*p* = 1.000). There was no significant difference in seizure rate (MSR 33% vs. SRT 5%; OR 1.77, 95% CI 0.57–6.59; *p* = 0.300). Tumor types were equally distributed (*p* = 0.700).

**Table 2:** Comparison of baseline characteristics and outcome parameters in MSR and SRT

Characteristic	SRT^1^n=24	MSR^1^n=109	p-value^2^	OR (95% CI)
Age at treatment	60 (54–75)	66 (57–75)	0.2	
*Cancer type*			0.7	
Lung cancer	10 (42%)	59 (54%)		
Breast Cancer	4 (17%)	8 (7.3%)		
Malignant Melanoma	2 (8.3%)	6 (5.5%)		
GIT	3 (13%)	11 (10%)		
Urothelial	1 (4.2%)	2 (1.8%)		
GYN	0 (0%)	(0.9%)		
RCC	3 (13%)	8 (7.3%)		
NET	0 (0%)	1 (0.9%)		
Prostate	0 (0%)	2 (1.8%)		
Other	1 (4.2%)	10 (9.2%)		
CUP	0 (0%)	1 (0.9%)		
Number intracranial metastases	2 (2–5)	1 (1–3)	0.008*	
Hemorrhagic metastases	0 (0%)	14 (13%)	0.073	¥ (0.77–¥)
Extracerebral metastases	16 (67%)	35 (32%)	0.002**	0.24 (0.08– 0.66)
Volume [cm^3^]	1 (0–3)	3 (1–6)	0.002**	
BM first diagnosis of cancer	6 (25%)	45 (41%)	0.2	2.1 (0.72–6.98)
Prior systemic treatment	15 (63%)	40 (37%)	0.038*	0.36 (0.13–0.98)
Time diagnosis to treatment [days]	864 (77–1,286)	98 (7–947)	0.023*	
Clinical performance pre				
KPS pre-treatment	70 (60–80)	80 (70–90)	0.10	
Worst MRC pre-treatment	4 (4–5)	4 (3–4)	0.015*	
Seizure pre-treatment	5 (22%)	36 (33%)	0.3	1.77 (0.57–6.59)
Ambulation pre-treatment	18 (78%)	85 (79%)	>0.9	1.03 (0.27–3.29)
CCI	1 (0–1)	0 (0–1)	0.8	
Clinical performance post				
KPS post-treatment	70 (60–80)	80 (70–90)	0.015*	
Worst MRC post-treatment	4 (4–5)	4 (3–5)	0.13	
Ambulation post-treatment	11 (58%)	74 (70%)	0.3	
Motor function post-treatment			0.4	
Stable	14 (61%)	62 (57%)		
Improved	3 (13%)	27 (25%)		
Worsened	6 (26%)	20 (18%)		
Length of hospital stay [days]	9 (7–21)^ɑ^	11 (7–16)	0.8	
Survival from treatment [months]	4 (0–16)	4 (0–13)	>0.9	
Clinical performance 6-month FU^β^				
Worst MRC at 6 months	4 (4–5)		0.2	
Motor function at 6 months			0.063	
Stable	8 (73%)	24 (45%)		
Improved	1 (9.1%)	24 (45%)		
Worsened	2 (18%)	5 (9.4%)		
Seizure at 6 months	1 (11%)	7 (13%)	>0.9	1.24 (0.13–62.99)
KPS at last FU	60 (30–70)	70 (40–80)	0.15	

^1^Median (Q1–Q3); n (%)

^2^Wilcoxon rank sum test; Fisher's exact test

^ɑ^n=17, seven patients treated on outpatient basis

^β^Of all data available at FU, SRT n=11 and MSR n=60

Multivariable logistic regression analyses showed that higher preoperative KPS increased likelihood of surgical treatment (OR 1.06 per point, *p* = 0.008), whereas better motor function was associated with a lower likelihood of surgery (OR 0.45 per point, *p* = 0.004).

### Neurological outcome of treatment groups

**Fig. 3:**
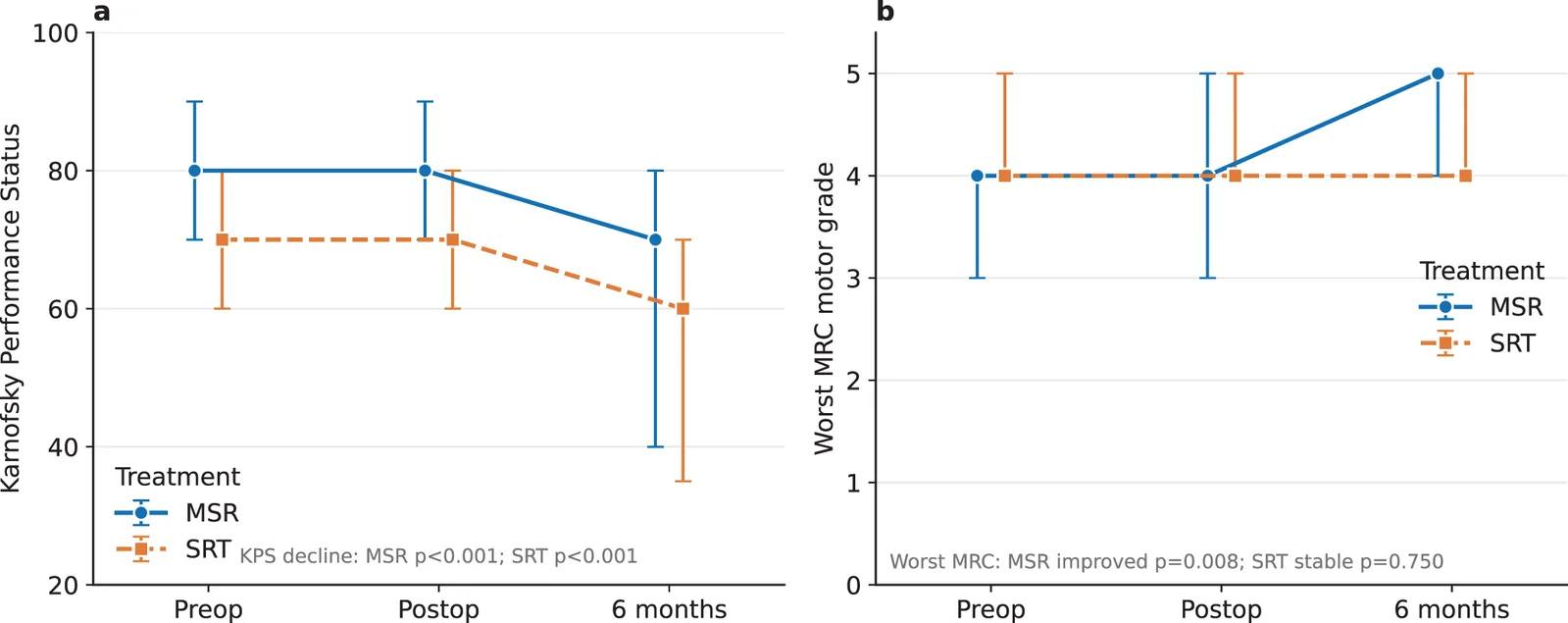
Longitudinal trajectories of functional outcome measures in MSR and SRT cohort in both treatment cohorts, KPS declined significantly between baseline and 6-month FU (MSR: *p* < 0.001; SRT: *p* < 0.001). Worst motor grade remained stable in SRT (*p* = 0.750) and improved significantly in MSR (*p* = 0.008). Values presented as median, whisker presenting IQR

Post-treatment, median motor grades were comparable in both cohorts (*p* = 0.130) and did not change significantly compared to pre-treatment in both cohorts (MSR: *p* = 0.285, SRT *p* = 0.271). At 6-month FU, median motor function was likewise similar (*p* = 0.200): median MRC was 5 (IQR 4–5) in the MSR cohort and 4 (IQR 4–5) in the SRT cohort. Longitudinal analyses showed a significant improvement in median MRC in the MSR cohort (*p* = 0.008) (Fig. 3).

Post-treatment Karnofsky-Index was 80 (IQR 70–90) in the MSR cohort and 70 (IQR 60–80) in the SRT cohort (*p* = 0.015). Motor function post-treatment did not differ significantly between groups (*p* = 0.400). In the MSR cohort, approximately 25% improved, 57% remained stable, and 18% deteriorated, compared to 13%, 61%, and 26% in the SRT cohort, respectively. At 6 months, data was available in 60 MSR patients and 11 SRT patients. Improvement was observed in 45% in the MSR group and 9.1% in the SRT group, while stability was seen in 45% and 73%, and deterioration in 9.4% and 18% (*p* = 0.063). These differences did not reach statistical significance, although a trend toward higher improvement rates following surgery was noted. Postoperatively, ambulation was maintained in 70% patients following surgery and in 58% after radiotherapy (OR 1.67, 95% CI 0.53–5.08, *p* = 0.300).

### Survival, local control, and adjuvant treatment

At last available FU, 27 of 150 patients with documented survival status had died (18%), with comparable mortality in the MSR (19/108, 18%) and SRT (4/23, 17%) cohorts. Because only 18% of patients had died at the time of analysis, the median overall survival from cancer diagnosis was not reached. Descriptively, observed survival from cancer diagnosis was longer in the SRT than in the MSR cohort (median 38 months, IQR 24–62 versus 15 months, IQR 2–44; *p* = 0.06). Cause of death (central nervous system versus systemic progression) was not systematically recorded. Local recurrence of the index BM was documented in 7 of 109 surgically treated patients (6.4%). Adjuvant post-operative radiotherapy was administered in 86 of 91 MSR patients with available data (95%), consistent with the current standard of care. Systemic treatment was recorded before (MSR 37%, SRT 63%) and after local therapy (including immunotherapy; MSR 68%, SRT 71%).

In multivariable Firth logistic regression analysis adjusted for treatment modality, pre-treatment motor deficit was not associated with neurological deterioration at 6-month FU (OR 0.73, 95% CI 0.44–1.31, *p* = 0.255). Likewise, treatment modality itself was not independently associated with neurological deterioration (OR 0.31, 95% CI 0.05–2.07, *p* = 0.210). In sensitivity analyses adjusted for treatment modality, neither preoperative KPS (OR 1.02, 95% CI 0.95–1.10, *p* = 0.648),CCI (OR 1.07, 95% CI 0.54–1.81, *p* = 0.806) nor tumor volume (OR 1.01, 95% CI 0.96–1.04, *p* = 0.666) were associated with neurological deterioration at 6-month FU.

### Sub-cohort analyses of high-risk vs. low-risk patients in MSR

Patients with characteristics according to pre-specified criteria (Table 1) which were unfavorable of MSR within the MSR cohort were classified as high-risk patients (*n* = 66) and subsequently compared with low-risk patients (*n* = 43). Median age was 65.5 years (IQR 56–74) and 67 years (IQR 59–76) respectively.

Pre-treatment MRC was 4 (IQR 3–4) in the low-risk and 4 (IQR 3–4) in the high-risk group (*p* = 0.561). Simultaneously, post-treatment MRC was 4 (IQR 3–5) in both groups (*p* = 0.444). Six months FU was available in 18 low-risk patients and 24 high-risk patients. Median motor grades were 5 (IQR 4–5) and 5 (IQR 4–5) (*p* = 0.306), respectively. Ambulation was similarly preserved in both groups pre-treatment (86.0% vs. 81.8%; OR 1.37, 95% CI 0.45–4.17; *p* = 0.600) as well as post-treatment (79.1% vs. 77.3%; OR 1.12, 95% CI 0.39–3.18; *p* = 0.840).

### Crossover cohort

Seventeen patients initially treated with radiotherapy underwent subsequent surgical resection due to new neurological impairment or deterioration, respectively. Mean age was 66 years (IQR 55–71). Primary cancer was lung cancer in 41.2%. Median time from cancer diagnosis to surgery of the index metastasis was 728 days (IQR 414–1889). Prior to surgery, all patients were neurologically impaired with a median motor function of 4 (IQR 2–4), and 58.8% were ambulatory. At discharge, median motor function was 3 (IQR 0–5), with 12.5% of patients improving, 62.5% remaining neurologically stable, and 25.0% deteriorating. At 6-month FU (*n* = 6), median motor function improved to 4.5 (IQR 4–5), with 33% of patients remaining stable, 67% of patients demonstrating neurological improvement and no observed deterioration.

Compared to the initial MSR cohort, there were significant differences in the presence of extracerebral metastases (69% vs. 32% in MSR, OR 4.59, 95% CI 1.35–18.19; *p* = 0.010) and time from diagnosis to surgery (728 days vs. 98 days, *p* = 0.007). Furthermore, BMs were significantly less likely to be the initial manifestation leading to the diagnosis of the primary cancer in the crossover cohort compared with the MSR group (12% vs. 41%, OR 0.19, 95% CI 0.02–0.89; *p* = 0.029).

## Discussion

This study assesses treatment selection and functional outcomes in patients with central BM. It focuses on patient-specific factors and their influence on the choice of therapy and on functional outcomes.

Our retrospective analysis compared baseline clinical parameters between the MSR and SRT cohorts. When discussing the appropriate individual treatment for any patient with central BM, clinical performance is frequently considered. However, there are currently no dedicated guidelines addressing patient-specific characteristics or defining functional cutoffs for treatment allocation in centrally located BM. Consequently, treatment decisions are largely based on interdisciplinary clinical judgement and physician experience. In our cohort KPS pre-treatment was comparable in both cohorts.

We found, however, that longer disease history, prior systemic treatment, higher intracranial and extracranial burden were associated with treatment by SRT. Median tumor volume was significantly smaller in this group. Multivariable regression further demonstrated that treatment allocation was strongly influenced by disease burden and neurological status. The lack of significant differences in univariate comparisons likely reflects overlapping distributions between groups, whereas multivariable analysis accounts for confounding factors and reveals independent predictors of treatment selection. Overall, these findings suggest that patients selected for surgery were generally in good systemic condition while simultaneously exhibiting neurological deficits. This observation is consistent with prior studies and the general assumption that the primary aim of tumor resection is functional preservation [30, 31]. In addition, reduction of intracranial tumor burden may decrease seizure rate and prolong overall survival [6], thereby supporting MSR even in selected asymptomatic patients. Conversely, because patients with metastatic disease will most likely succumb to systemic rather than intracranial disease progression, some clinicians consider brain surgery an unnecessary procedural risk [32]. In line with this assumption, our data demonstrated a significant decline in KPS over time in both cohorts, likely reflecting progressive systemic disease burden despite local intracranial treatment. However, post-treatment KPS remained significantly higher in the MSR cohort. While not statistically significant, first-time diagnosis of metastatic disease was more frequent in the MSR group though.

General prognostic frameworks, such as the Recursive Partitioning Analysis classification developed by the Radiation Therapy Oncology Group, as well as contemporary guideline recommendations, provide general treatment algorithms for BM based on factors including tumor size, age and functional status [2, 14, 18]. Pintea et al. reported that postoperative motor deterioration correlates with tumor size, suggesting that larger metastases may carry a higher risk of neurological decline [30]. Nevertheless, explicit recommendations for perirolandic BM, which are associated with particularly high risk of postoperative neurological impairment, remain lacking. In our cohort, preoperative motor function was not associated with postoperative neurological deterioration, indicating that neither severely symptomatic patients nor neurologically intact patients exhibited an increased risk of neurological decline following surgery. Furthermore, after adjustment for treatment modality, regression analyses demonstrated no association between pre-treatment overall clinical performance and neurological deterioration at 6-month FU. Collectively, these findings demonstrate that aggressive treatment strategies for centrally located BM may be justified and are not associated with inferior neurological outcomes. Importantly, technical advances in surgery as well as radiotherapy such as TMS mapping have substantially improved procedural safety and functional preservation in recent years [17, 25]. Durable local control is also relevant for preventing secondary neurological deterioration from local recurrence or leptomeningeal disease [12]. In our MSR cohort, local recurrence was low at 6.4%, possibly reflecting the routine use of adjuvant cavity radiotherapy in 95% of surgically treated patients. Thus, microsurgical resection within a multimodal treatment concept may provide effective local control even for motor cortex metastases. Future studies should prospectively evaluate local recurrence rates in both treatment groups.

Systemic oncological therapies continue to evolve, leading to prolonged survival in patients with metastatic disease. Consequently, the challenge of selecting the most appropriate treatment strategy for older and heavily pretreated patients will become increasingly relevant in neuro-oncological practice. One important advantage of surgery in this context is the possibility of tissue sampling for histopathological and molecular analysis. This may guide targeted systemic therapy.

To further investigate whether patients with prolonged disease history and higher intra- as well as extracranial tumor burden experienced worse post-treatment outcomes, we developed a risk score to dichotomize the MSR cohort. Functional outcomes both postoperatively and at 6-month FU were comparable between low-risk and high-risk subgroups. These findings suggest that surgery may remain feasible and may be considered in patients presenting with an unfavorable baseline profile. Notably, even severely impaired neurological function at time of either treatment initiation is not associated with neurological deterioration. Median survival in patients with BM varies substantially depending on cancer type making generalized statements regarding long-term FU difficult. This variability was reflected in our cohort, in which observed survival from cancer diagnosis differed markedly between cohorts (median 15 months in the MSR and 38 months in the SRT cohort), while the median overall survival was not reached at the current event rate. We therefore deliberately chose a 6-month FU as a primary endpoint to obtain meaningful longitudinal outcome data in a sufficient number of patients.

Consistent with current clinical practice, neurological deficits were less prevalent in the SRT cohort. Conversely, only 15% of surgically treated patients were neurologically asymptomatic at the time of intervention. In contrast to previous reports [30], and while median MRC improved significantly only in MSR, we did not observe a significant difference in neurological improvement rates between treatment cohorts. This may partly reflect unequal group sizes and limited statistical power.

Notably, a subgroup of patients initially underwent SRT/WBRT for one or more index metastases before later requiring surgical intervention. In all these patients, the indication for surgery was the development of new and acute neurological deficits. Although postoperative KPS tended to be lower in these crossover patients compared with patients undergoing upfront MSR, this trend did not reach significance, most likely due to limited sample size.

In general, this study is limited by its retrospective and monocentric character. Regression analyses were conducted with limited parameters to avoid overfitting both cohorts. Limited FU periods lead to wide confidence intervals and hinder reliable interpretation, which, in conjunction with unequal group sizes, render this an observational study and do not infer causality. It should further be noted that the risk score was developed as an exploratory post hoc analysis. It was not externally validated and did not discriminate functional outcomes and should therefore be regarded as hypothesis-generating. Data on local control were limited to documented recurrence of the index BM, whereas radiographic response and leptomeningeal dissemination were not systematically assessed; likewise, cause-specific mortality, radiotherapy dose regimens, and specific systemic treatments (e.g. immunotherapeutic agents) and histopathology in the crossover cohort (viable tumor versus radiation necrosis) were not available for analysis. Peritumoral edema, which may contribute to symptom aggravation, was not included in the volumetric measurements. Finally, the small SRT cohort, its divergent baseline characteristics, and high crossover rate limit the validity of the direct treatment comparisons.

## Conclusion

Although treatment guidelines for BM exist, specific recommendations for centrally located metastases remain lacking. Factors such as tumor size, patient age, and clinical performance status may aid treatment selection. However, in daily clinical practice, therapeutic decisions are frequently individualized and largely guided by clinical experience and the patient’s overall disease trajectory.

In our cohort, patients selected for MSR exhibited superior motor deficit improvements on FU, particularly in selected high-risk patients. In contrast, in our limited cohort SRT remained the preferred treatment strategy in more frail patients with impaired overall performance status.

## Electronic supplementary material

Below is the link to the electronic supplementary material.

Supplementary material 1

## Data Availability

The anonymized datasets generated during and/or analyzed during the current study are available from the corresponding author on reasonable request.

## Abbreviation

BM: Brain metastases
CCI: Charlson Comorbidity Index
CUP: Cancer of unknown primary
FU: Follow-up
GIT: Gastrointestinal cancer
GYN: Gynecological cancer
MRC: Medical research council muscle strength grading scale
MSR: Microsurgical resection
NET: Neuroendocrine tumor
RCC: Renal cell cancer
SRT: Stereotactic radiotherapy
TMS: Transcranial motor stimulation
WBRT: Whole brain radiotherapy

## References

[1] Patchell RA (1991) Brain metastases. Neurol Clin 9(4):817–824. 10.1016/S0733-8619(18)30250-0 1758427

[2] Ene CI, Ferguson SD (2022) Surgical management of brain metastasis: challenges and nuances. Front Oncol 12:847110. 10.3389/fonc.2022.847110 PMC8963990 35359380

[3] Patchell RA, Tibbs PA, Walsh JW, Dempsey RJ, Maruyama Y, Kryscio RJ, Markesbery WR, Macdonald JS, Young B (1990) A randomized trial of surgery in the treatment of single metastases to the brain. N Engl J Med 322(8):494–500. 10.1056/NEJM199002223220802 2405271

[4] Rhun EL, Guckenberger M, Smits M, Dummer R, Bachelot T, Sahm F, Galldiks N, de Azambuja E, Berghoff AS, Metellus P, Peters S, Hong Y-K, Winkler F, Schadendorf D, van den Bent M, Seoane J, Stahel R, Minniti G, Wesseling P, Weller M, Preusser M (2021) EANO–ESMO clinical practice guidelines for diagnosis, treatment and follow-up of patients with brain metastasis from solid tumours. Ann Oncol 32(11):1332–1347. 10.1016/j.annonc.2021.07.016 34364998

[5] Soffietti R, Abacioglu U, Baumert B, Combs SE, Kinhult S, Kros JM, Marosi C, Metellus P, Radbruch A, Villa Freixa SS, Brada M, Carapella CM, Preusser M, Le Rhun E, Rudà R, Tonn JC, Weber DC, Weller M (2017) Diagnosis and treatment of brain metastases from solid tumors: guidelines from the European Association of Neuro-Oncology (EANO). Neuro-oncology 19(2):162–174. 10.1093/neuonc/now241 PMC5620494 28391295

[6] Goldberg M, Kraus LM, Vatcheva C, Bernhardt D, Combs SE, Negwer C, Meyer B, Wagner A (2025) Evaluating the impact of index metastasis resection in patients with multiple brain metastases. Cancers 17(20):3281. 10.3390/cancers17203281 PMC12563598 41154339

[7] Schackert G, Lindner C, Petschke S, Leimert M, Kirsch M (2013) Retrospective study of 127 surgically treated patients with multiple brain metastases: indication, prognostic factors, and outcome. Acta Neurochir (Wien) 155(3):379–387. 10.1007/s00701-012-1606-8 23314988

[8] Bindal RK, Sawaya R, Leavens ME, Lee JJ (1993) Surgical treatment of multiple brain metastases. J Neurosurg 79(2):210–216. 10.3171/jns.1993.79.2.0210 8331402

[9] Salvati M, Tropeano MP, Maiola V, Lavalle L, Brogna C, Colonnese C, Frati A, D’Elia A (2018) Multiple brain metastases: a surgical series and neurosurgical perspective. Neurol Sci 39(4):671–677. 10.1007/s10072-017-3220-2 29383618

[10] Lin J, Kaiser Y, Wiestler B, Bernhardt D, Combs SE, Delbridge C, Meyer B, Gempt J, Aftahy AK (2023) Cytoreduction of residual tumor burden is decisive for prolonged survival in patients with recurrent brain metastases—retrospective analysis of 219 patients. Cancers 15(20):5067. 10.3390/cancers15205067 PMC10605169 37894435

[11] Altieri R, Corvino S, La Rocca G, Cofano F, Melcarne A, Garbossa D, Barbarisi M (2024) Clinical implication of brain metastases en-bloc resection: surgical technique description and literature review. JPM 14(11):1110. 10.3390/jpm14111110 PMC11596013 39590602

[12] Suki D, Hatiboglu MA, Patel AJ, Weinberg JS, Groves MD, Mahajan A, Sawaya R (2009) Comparative risk of leptomeningeal dissemination of cancer after surgery or stereotactic radiosurgery for a single supratentorial solid tumor metastasis. Neurosurgery 64(4):664. 10.1227/01.NEU.0000341535.53720.3E 19197219

[13] Obermueller T, Schaeffner M, Gerhardt J, Meyer B, Ringel F, Krieg SM (2014) Risks of postoperative paresis in motor eloquently and non-eloquently located brain metastases. BMC Cancer 14(1):21. 10.1186/1471-2407-14-21 PMC3899614 24422871

[14] Zuo F, Hu K, Kong J, Zhang Y, Wan J Surgical management of brain metastases in the perirolandic region. Front Oncol 10. 10.3389/fonc.2020.572644. 26 October 2020 PMC7649351 33194673

[15] Sanmillan JL, Fernández-Coello A, Fernández-Conejero I, Plans G, Gabarrós A (2017) Functional approach using intraoperative brain mapping and neurophysiological monitoring for the surgical treatment of brain metastases in the central region. JNS 126(3):698–707. 10.3171/2016.2.JNS152855 27128588

[16] Obermueller T, Schaeffner M, Shiban E, Droese D, Negwer C, Meyer B, Ringel F, Krieg SM (2015) Intraoperative neuromonitoring for function-guided resection differs for supratentorial motor eloquent gliomas and metastases. BMC Neurol 15(1):211. 10.1186/s12883-015-0476-0 PMC4618356 26487091

[17] Krieg SM, Schäffner M, Shiban E, Droese D, Obermüller T, Gempt J, Meyer B, Ringel F (2013) Reliability of intraoperative neurophysiological monitoring using motor evoked potentials during resection of metastases in motor-eloquent brain regions: clinical article. J Neurosurg 118(6):1269–1278. 10.3171/2013.2.JNS121752 23521547

[18] Rossetto M, Ciccarino P, Lombardi G, Rolma G, Cecchin D, Della Puppa A (2016) Surgery on motor area metastasis. Neurosurg Rev 39(1):71–78. 10.1007/s10143-015-0648-9 26178239

[19] Magill ST, Han SJ, Li J, Berger MS (2018) Resection of primary motor cortex tumors: feasibility and surgical outcomes. J Neurosurg 129(4):961–972. 10.3171/2017.5.JNS163045 29219753

[20] Luther N, Kondziolka D, Kano H, Mousavi SH, Flickinger JC, Lunsford LD (2013) Motor function after stereotactic radiosurgery for brain metastases in the region of the motor cortex: clinical article. JNS 119(3):683–688. 10.3171/2013.6.JNS122081 23870018

[21] Tarapore PE, Picht T, Bulubas L, Shin Y, Kulchytska N, Meyer B, Berger MS, Nagarajan SS, Krieg SM (2016) Safety and tolerability of navigated TMS for preoperative mapping in neurosurgical patients. Clin Neurophysiol 127(3):1895–1900. 10.1016/j.clinph.2015.11.042 26762952

[22] Diehl CD, Rosenkranz E, Schwendner M, Mißlbeck M, Sollmann N, Ille S, Meyer B, Combs SE, Krieg SM (2022) Dose reduction to motor structures in adjuvant fractionated stereotactic radiotherapy of brain metastases: nTMS-derived DTI-Based motor fiber tracking in treatment planning. Cancers 15(1):282. 10.3390/cancers15010282 PMC9818359 36612277

[23] Colman J, Mancini L, Manolopoulos S, Gupta M, Kosmin M, Bisdas S (2022) Is diffusion tensor imaging-guided radiotherapy the new state-of-the-art? A review of the current literature and technical insights. Appl Sci 12(2):816. 10.3390/app12020816

[24] Krieg SM, Shiban E, Buchmann N, Meyer B, Ringel F (2013) Presurgical navigated transcranial magnetic brain stimulation for recurrent gliomas in motor eloquent areas. Clin Neurophysiol 124(3):522–527. 10.1016/j.clinph.2012.08.011 22986282

[25] Krieg SM, Sabih J, Bulubasova L, Obermueller T, Negwer C, Janssen I, Shiban E, Meyer B, Ringel F (2014) Preoperative motor mapping by navigated transcranial magnetic brain stimulation improves outcome for motor eloquent lesions. Neuro-Oncol 16(9):1274–1282. 10.1093/neuonc/nou007 PMC4136889 24516237

[26] Karnofsky DA, Burchenal JH (1949) The clinical evaluation of chemotherapeutic agents in cancer. In: Macleod CM (ed) Evaluation of chemotherapeutic agents. New York Academy of Medicine, New York

[27] Singhal S, Gill M, Srivastava C, Gupta D, Kumar A, Kaushik A, Semwal M (2020) Simplifying tumor volume estimation from linear dimensions for intra-cranial lesions treated with stereotactic radiosurgery. J Med Phys 45(4):199–205. 10.4103/jmp.JMP_56_20 PMC8074724 33953494

[28] Charlson ME, Pompei P, Ales KL, MacKenzie CR (1987) A new method of classifying prognostic comorbidity in longitudinal studies: development and validation. J Chronic Dis 40(5):373–383. 10.1016/0021-9681(87)90171-8 3558716

[29] Firth D (1993) Bias reduction of maximum likelihood estimates. Biometrika 80(1):27–38. 10.1093/biomet/80.1.27

[30] Pintea B, Baumert B, Kinfe TM, Gousias K, Parpaley Y, Boström JP (2017) Early motor function after local treatment of brain metastases in the motor cortex region with stereotactic radiotherapy/radiosurgery or microsurgical resection: a retrospective study of two consecutive cohorts. Radiat Oncol Lond Engl 12(1):177. 10.1186/s13014-017-0917-6 PMC5683312 29132382

[31] Schödel P, Jünger ST, Wittersheim M, Reinhardt HC, Schmidt N-O, Goldbrunner R, Proescholdt M, Grau S (2020) Surgical resection of symptomatic brain metastases improves the clinical status and facilitates further treatment. Cancer Med 9(20):7503–7510. 10.1002/cam4.3402 PMC7571801 32858763

[32] Gupta S, Dawood H, Giantini Larsen A, Fandino L, Knelson EH, Smith TR, Lee EQ, Aizer A, Dunn IF, Bi WL Surgical and peri-operative considerations for brain metastases. Front Oncol 11. 10.3389/fonc.2021.662943. 5 May 2021 PMC8131835 34026641

