# Supplementary material for: Treatment selection and functional outcomes in motor cortex metastases
Source: J Neurooncol. 2026 Aug 17;179(2):41. doi: 10.1007/s11060-026-05758-2 (PMC13481405; doi:10.1007/s11060-026-05758-2)
Supplement: Supplementary file 1 [file 11060_2026_5758_MOESM1_ESM.pdf]

	Maximum score
Intracranial tumor burden (solitary vs. multifocal)	1
Prior systemic treatment	1
Prevalence of extracerebral metastases	1
Time from cancer diagnosis to treatment > 98 days*	1
	**4**
